# Cardiac blood vessels and irreversible electroporation: findings from pulsed field ablation

**DOI:** 10.20517/2574-1209.2023.80

**Published:** 2024-01-30

**Authors:** Ikeotunye Royal Chinyere, Shumpei Mori, Mathew D. Hutchinson

**Affiliations:** 1 Sarver Heart Center, University of Arizona, Tucson, AZ 85724, USA; 2 Banner University Medicine, Banner Health, Tucson, AZ 85719, USA; 3 UCLA Cardiac Arrhythmia Center, UCLA Health System, David Geffen School of Medicine at UCLA, Los Angeles, CA 90095, USA

**Keywords:** Spasm, stenosis, coronary, pulmonary, electrophysiology, arrhythmia, safety, catheter

## Abstract

The clinical use of irreversible electroporation in invasive cardiac laboratories, termed pulsed field ablation (PFA), is gaining early enthusiasm among electrophysiologists for the management of both atrial and ventricular arrhythmogenic substrates. Though electroporation is regularly employed in other branches of science and medicine, concerns regarding the acute and permanent vascular effects of PFA remain. This comprehensive review aims to summarize the preclinical and adult clinical data published to date on PFA’s effects on pulmonary veins and coronary arteries. These data will be contrasted with the incidences of iatrogenic pulmonary vein stenosis and coronary artery injury secondary to thermal cardiac ablation modalities, namely radiofrequency energy, laser energy, and liquid nitrogen-based cryoablation.

## INTRODUCTION

### Preface

This review article begins by introducing the history of irreversible electroporation before describing its mechanism of action and clinical application within cardiac electrophysiology. A brief summary of the vascular damage associated with thermal cardiac ablation is provided before atrial pulsed field ablation (highlighting preclinical and clinical pulmonary vein findings) and ventricular pulsed field ablation (highlighting preclinical and clinical coronary artery findings) are comprehensively reviewed and contrasted with the aforementioned thermal ablation outcomes.

### Historical perspective

The investigation of therapeutic *in vivo* cardiac electroporation first appeared in the scientific literature in the year 2007^[1]^. *In vitro* cardiomyocyte electroporation was described as early as 1987, as the molecular complications of defibrillation and cardioversion were being elucidated^[2]^. This was many years after cell biology applications for electroporation were already regularly utilized in research laboratories. Clinically, oncology was the first medical discipline to utilize therapeutic electroporation, with literature dating back to the year 1994^[3]^.

### Mechanism of action and clinical application

Electroporation is defined as the application of brief supra-physiologic electric field pulses to a cell membrane such that the resulting voltage gradient overwhelms the phospholipid bilayer’s electrical capacitance^[4]^, structurally destabilizing it via intramembrane current flow^[5]^. Microscopic zones of fluid vaporization lead to the spontaneous development of unstable pores of various sizes. This process can be performed without macroscopic heat generation, assuming a relatively low voltage per centimeter or low pulse duration/frequency, and subsequently minimal resistance from macroscopic current flow^[6,7]^.

The ability to tune the pulse characteristics and field characteristics for irreversible electroporation allows for the targeting of specific tissue types. To this end, a “single shot” myocardium-targeting technique is being increasingly employed that minimizes heat generation and decreases case time, though multiple pulses are likely optimal for sufficient electrical isolation^[8]^. These pores increase the membrane permeability to a recoverable (reversible) or lethal (irreversible) degree, depending on their quantity, distribution, and diameter^[9]^. The necessity of a membrane elucidates irreversible electroporation’s ability to spare non-cell-based organic materials such as elastin and collagen, preserving extracellular matrices and subsequent tissue architecture while destroying cellular residents with a susceptible membrane dielectric constant contained within the electric field. Due to their epicardial location, it is thought that autonomic neuron ganglia/terminals may also be preserved with endocardial irreversible electroporation if not contained in the local electric field or oriented properly relative to plane(s) of the electric field^[10-12]^.

The ability to apply sufficiently large electric field pulses using modern endovascular catheter techniques^[13,14]^ facilitated the translation of irreversible electroporation to clinical cardiac electrophysiology for arrhythmogenic substrate management, termed pulsed field ablation (PFA). These electric field pulses can be applied in either a monophasic or biphasic waveform via either a unipolar or bipolar electrode configuration, with the biphasic-bipolar approach having the highest effect-specificity for irreversible plasma membrane pore creation, and allowing for precise lesion areas^[13]^ with a wider margin of error regarding contact force^[15]^ relative to the margin for thermal ablation techniques. The risk of unintentional arrhythmia induction by PFA during a vulnerable phase of the cardiac action potential is mitigated by gating the generator output to the surface electrocardiogram^[16,17]^. The presence of metal-containing biomaterials from an interventional cardiology case may amplify the pulsed electric field, though only computational data is currently available^[18]^.

Though no clinical guidelines presently exist to govern the application of PFA by electrophysiologists in the United States or in Europe, the consensus of early adopters equates PFA with all prior forms of catheter ablation. Thus, in the European Union where a number of PFA systems have achieved CE certification, PFA may be pursued after failed rhythm control with pharmacologic therapy for patients suffering from atrial fibrillation (AFib)^[19]^. The advantage of PFA over thermal ablation modalities has become evident when anatomically challenging targets, due to thermal catheter technical limitations or neighboring temperature-sensitive structures, are successfully navigated without an increase in complications or a change in short-term outcomes.

### Blood vessel injury from thermal cardiac ablation modalities

**Figure 1.**
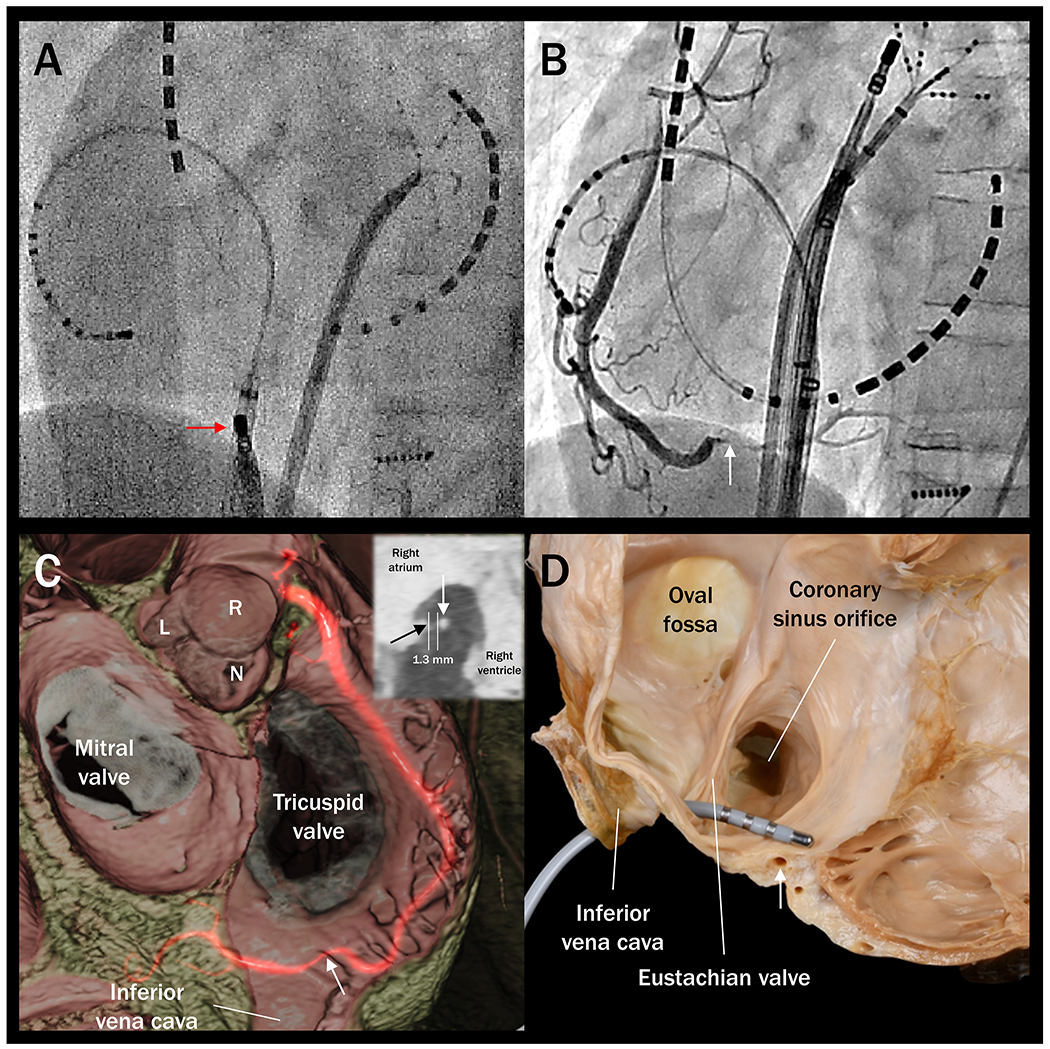
Right coronary artery injury after radiofrequency ablation of the cavotricuspid isthmus. (A) Fluoroscopic radiograph (left anterior oblique view) depicts the ablation catheter (red arrow) placed at 6 o’clock position on the cavotricuspid isthmus. During the procedure, the patient developed chest pain with ST- segment elevation in the inferior surface electrocardiogram leads. (B) Emergent coronary angiography revealed near-total occlusion of the distal right coronary artery (white arrow). (C) Follow-up cardiac computed tomography post-revascularization revealed the location of the stenotic segment of the right coronary artery (white arrow) behind the pectinate muscles. This volume-rendered virtual dissection image is viewed from the right posterior oblique view and cranial direction. Multiplanar reconstruction image (C) top-right insertion viewed from the right anterior oblique direction reveals the distance between the right atrial vestibule (black arrow) and the affected right coronary artery (white arrow) is 1.3 millimeters at the pocket beneath the pectinate muscle. (D) Dissection image viewed from the right anterior oblique direction exhibits the proximity of the catheter tip to the distal right coronary artery (white arrow) during cavotricuspid isthmus ablation. L: left coronary aortic sinus; N: non-coronary aortic sinus; R: right coronary aortic sinus; mm: millimeters.

**Figure 2.**
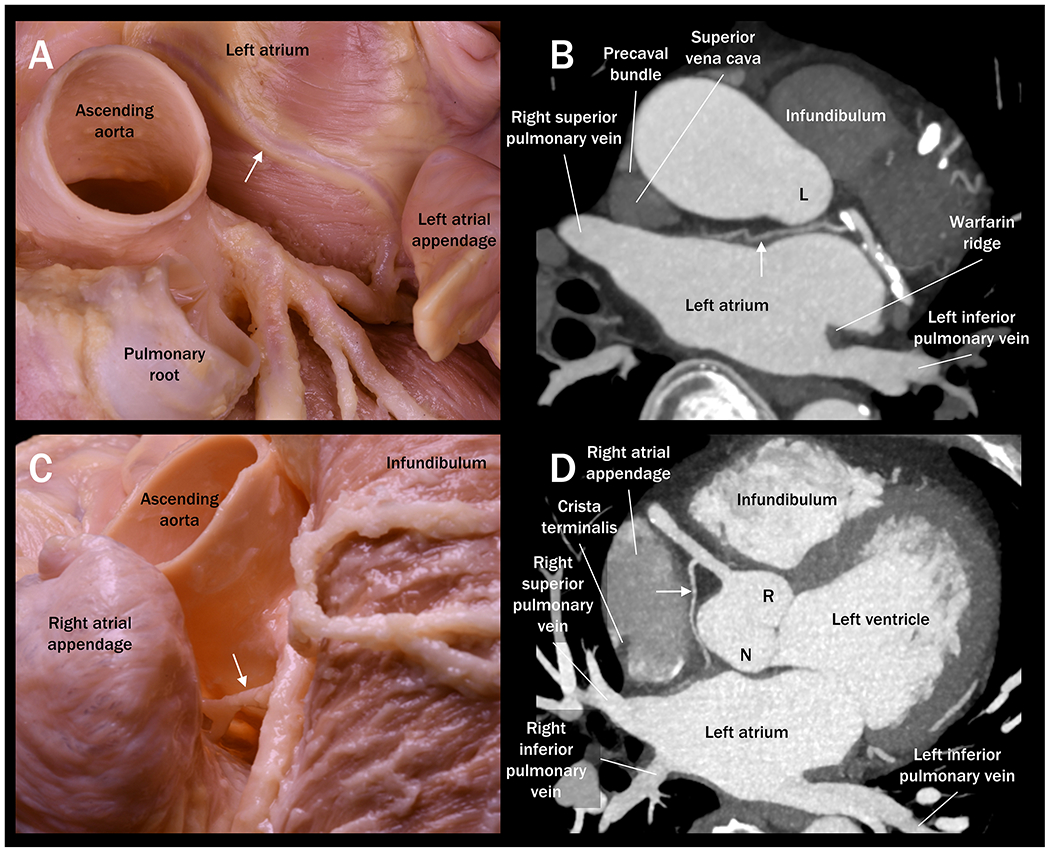
Anatomy of the left and right sinuatrial nodal arteries. (A) High-resolution dissection-enhanced photograph of the left sinuatrial nodal artery (white arrow), which originates from the left circumflex artery and runs along the anterior wall of the left atrium within the Bachmann’s bundle. (B) Multiplanar reconstruction image from the cardiac computed tomography shows the left sinuatrial nodal artery (white arrow) running similar course, suggesting a potential risk of injury of this artery during radiofrequency catheter ablation of the anterior wall of the left atrium. (C) High-resolution dissection-enhanced photograph shows the right sinuatrial nodal artery (white arrow) running between the right atrial appendage and the aortic root. Note that epicardial fat filling this region was thoroughly removed to expose this artery. (D) Multiplanar reconstruction image from the cardiac computed tomography shows the right sinuatrial nodal artery (white arrow) running similar course, suggesting a potential risk of injury of this artery during radiofrequency catheter ablation of the medial wall of the right atrial appendage. L: left coronary aortic sinus; N: non-coronary aortic sinus; R: right coronary aortic sinus.

The utilization of cardiac catheters to rapidly facilitate local tissue temperature changes (heat for laser/radiofrequency energy and cold for liquid nitrogen-based cryoablation) and disrupt arrhythmogenic substrates carries the risk of unintended damage to the tissue neighboring the lesion [Figure 1]. Unlike PFA, these thermal ablation techniques destroy all cellular and extracellular tissue components via denaturing. Literature exists that proposes a porous media theory to describe and predict thermal ablation of myocardium^[20-22]^. Due to the compact mediastinal anatomy [Figure 2] and catheter movement due to cardiac activity or respiratory motion, tissues at risk for inadvertent or collateral damage include intracardiac tissues such as the conduction system and coronary arteries, and extracardiac tissues such as the phrenic nerve or esophagus.

Thermal ablation mechanisms-of-action are non-selective with regard to tissue/organ toxicity. Furthermore, heterogeneity in lesion depth due to variations in cardiac tissue density or adjacent blood flow-facilitated convective cooling can have a proarrhythmic effect via producing intra-myocardial pathways for aberrant conduction. Thermometric tools that approximate surface temperatures during ablation are employed in the cardiac electrophysiology lab, in addition to chilled/heated saline irrigations to protect off-target damage to adjacent essential structures and contact-force catheters. Nonetheless, thermal complications still occur^[23].^

**Figure 3.**
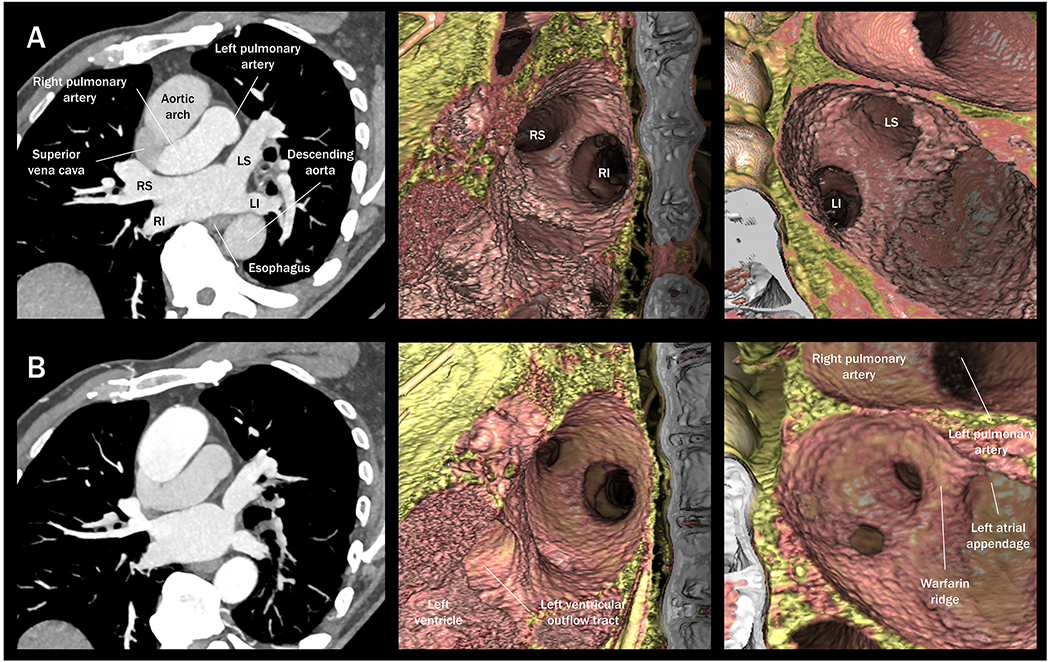
Pulmonary vein stenosis after balloon cryoablation. Cardiac computed tomography images (slab multiplanar reconstruction) before (A) and 8 months after (B) pulmonary vein isolation using a cryoablation balloon reveals moderate stenosis of the right superior, left superior, and left inferior pulmonary vein ostia. Middle panel images (left lateral view) and right panel images (right anterior oblique and caudal view) are volume-rendered virtual dissection images. LI: Left inferior pulmonary vein; LS: left superior pulmonary vein; RI: right inferior pulmonary vein; RS: right superior pulmonary vein.

Acquired pulmonary vein stenosis secondary to cardiac ablation is defined as the reduction in vein lumen diameter near the location of pulmonary vein ablation [Figure 3]. Grading of this stenosis, accomplished by dedicated cardiac non-invasive imaging, ranges from mild (less than 50% reduction in lumen diameter) to moderate, and severe (greater than 70% reduction in lumen diameter)^[24]^. Morbidity from this iatrogenic diagnosis can vary from minimal to severe symptom burdens, and though corrective procedures exist, success rates vary and repeat surgeries/percutaneous interventions may be necessary due to the dynamic resolution process^[25]^. The mechanism of this stenosis is attributed to damage to the intimal layer, causing intimal proliferation and/or fibrosis via myofibroblast activation^[26]^. The incidence of all grades of pulmonary vein stenosis from catheter-based endocardial ablations is reported to range from 21%^[27]^ to 42%^[28]^; however, multiple detection biases confound this statistic, particularly the number of and frequency of pulmonary vein imaging studies, given progression to persistent/stable pulmonary vein stenosis from the acute post-ablation stage is variable. Thankfully, the incidence of severe pulmonary vein stenosis with functionally limiting symptoms is negligible with increased operator awareness and modified protocols that minimize intra-vein energy delivery^[29]^.

Iatrogenic coronary artery vasospasm (time-limited vascular smooth muscle contraction to partial or complete lumen occlusion, usually less than one hour in duration) and post-ablation coronary artery stenosis (permanent restriction of lumen diameter) are markedly less common than acquired pulmonary vein stenosis, with an estimated incidence of < 1%^[30]^. This estimated incidence is likely an underestimate, given that post-ablation angiography is not routinely performed unless a patient’s symptoms warrant. Furthermore, aggregating cases that involve ablation near the coronary arteries would likely produce a greater incidence of coronary injury. The morbidity of coronary artery stenosis is similar to that of severe persistent pulmonary vein stenosis in that percutaneous and/or open interventions are necessary and may lead to subsequent repeat procedures. Implicated in both endocardial^[31,32]^ and epicardial^[33]^ ablation approaches, coronary artery stenosis can be graded using invasive angiographic techniques (Thrombolysis In Myocardial Infarction scale) or using non-invasive cardiac imaging techniques (example: contrast-enhanced computed tomography coronary angiogram).

## ATRIAL PULSED FIELD ABLATION

### Preface

It is necessary to acknowledge that multiple atrial ablation procedures implicate coronary arteries, including the right coronary artery during cavotricuspid isthmus ablation for right-sided flutter and the left coronary artery for perimitral ablation for left-sided flutter. Extremely limited data are available to discuss the coronary artery outcomes associated with pulsed field ablation for these particular arrhythmias. Subsequently, no discussion is warranted at this present time. However, we acknowledge the possibility of coronary artery effects during atrial pulsed field ablation, with the goal of increasing awareness for future investigation.

### Efficacy and pulmonary vein stenosis

Due to their well-established risk profiles and broad market penetration, thermal cardiac ablation techniques are the tools of choice for invasive cardiologists and cardiac surgeons aiming to modify atrial arrhythmogenic substrate. PFA has slowly gained enthusiasm, though not approved by the Food and Drug Administration at this time, and is being evaluated for its efficacy in decreasing arrhythmogenic burden in supraventricular tachycardia^[34]^, atrial tachycardia^[35,36]^, atrial flutter^[37]^, and atrial fibrillation^[38]^. In the non-pharmacologic management of atrial fibrillation, the plasticity of atrial substrate may necessitate multiple ablation procedures, including repeat pulmonary vein isolations or lesion extension to deal with postablation macroreentrant atrial tachycardia. The risk of pulmonary vein stenosis may correlate positively with the number of total thermal atrial fibrillation ablations^[39]^; however, parameter-optimized PFA has the theoretical advantage of being markedly toxic to cardiac myocytes, myofibroblasts, and other resident cardiac cell types due to their increased susceptibility. It is important to note the frequently encountered transient ST-segment elevation observed during PFA for pulmonary vein isolation secondary to ion movement disruption. No literature review to date has compiled both the preclinical and clinical outcomes regarding the prevalence of pulmonary vein stenosis with PFA versus the thermal alternatives.

### Preclinical pulmonary vein findings with atrial PFA

Though *in silico* studies on PFA exist^[40-42]^, the majority of preclinical PFA studies have been completed in *in vivo* animal models, both small^[43]^ and large. Of these *in vivo* studies, only the large animal studies were designed to recapitulate clinically relevant methodologies regarding atrial tachyarrhythmia catheter ablation techniques in the appropriate anatomic structures. Nine studies were identified, seven conducted in various swine models^[44-50]^ and two conducted in canine models^[51,52]^. Study designs and sample sizes varied widely [Table 1], and study durations ranged from 3 days^[47,52]^ to 3 months; however, no pulmonary vein stenosis was reported in any of these preclinical studies. Of note, the resolution of pulmonary vein injury secondary to thermal exposure is a slow, time-dependent process, and thus, these acute studies may underestimate the true incidence. Assessment of pulmonary vein diameter/function was predominately assessed via *postmortem* histopathology, though serial contrast- enhanced angiogram-based measurements were used as well^[44].^

### Clinical pulmonary vein findings with atrial PFA

Similar to the preclinical reports, no pulmonary vein stenosis was reported in any of the ten clinical publications utilizing PFA for atrial tachyarrhythmia catheter ablation^[53-62]^. The majority of these studies were completed by the same research group [Table 2], and most of the studies involved pre-ablation pulmonary vein imaging followed by repeat imaging at 3 months post-ablation. This study design is not appropriate to detect acute pulmonary vein reactions, but is meaningful with respect to identifying clinically relevant vascular changes. Though the sample size for each study was small (< 400 patients), the majority of the trials were conducted internationally, increasing the external validity of the findings.

### PFA pulmonary vein stenosis complication rate compared to thermal ablation

Due to the fact that no reports have been published describing pulmonary vein stenosis from PFA, this complication remains specific to thermal ablation techniques. Though non-thermal in mechanism-of-action, PFA is capable of producing heat^[6]^, specifically with high pulse cycle frequencies. Thus, pulmonary vein stenosis remains a theoretical complication of PFA as well. “Single shot” PFA applications reduce this theoretical risk to a negligible level.

## VENTRICULAR PULSED FIELD ABLATION

### Preface

It is necessary to acknowledge that pulsed field ablation has the possibility of affecting cardiac veins during ventricular ablation procedures. To date, no data exist describing these effects. For this reason, no discussion is warranted at this present time. Nonetheless, we acknowledge the possibility of cardiac vein effects during ventricular pulsed field ablation, with the goal of increasing awareness for future investigation.

### Efficacy and coronary artery spasm/stenosis

Applications for PFA include both atrial and ventricular arrhythmogenic substrate management. Though first described^[1]^ and clinically approved in Europe^[63]^ for atrial substrate modification, preclinical ventricular PFA data have suggested more favorable and homogeneous lesion characteristics in both normal and scarred ventricular myocardium^[64-67]^. However, additional preclinical and clinical evaluation is still necessary to clarify this potential advantage, relative to thermal catheter-based ablation techniques, namely radiofrequency energy. Theories attempting to explain this early data include a relatively high sensitivity of cardiomyocytes to PFA compared to the sensitivities of neighboring cardiac and non-cardiac (i.e., neurons, endothelial cells) cell types^[68]^. Though the details regarding the safety and durability of PFA continue to be elucidated in time, PFA may one day be a unique catheter-based electrophysiology tool that complements thermal energy in the electrophysiologist’s invasive armamentarium.

One of the safety questions that remains is how PFA affects ventricular cardiac vessels, specifically the epicardial coronary arteries. Irrespective of therapeutic efficacy, damage to the cells of any of the three muscular artery layers is to be avoided; otherwise, PFA would mitigate one arrhythmogenic risk but increase others. Any reactive changes in coronary arteries could lead to intimal hyperplasia and coronary lumen narrowing, smooth muscle hypertrophy and increased propensity to coronary vasospasm, or connective tissue disruptions leading to poor elasticity or compromised target vessels for surgical interventions such as coronary artery bypass grafting. Interestingly, early translational publications clearly documented irreversible electroporation’s ability to disrupt vascular smooth muscle cells and eradicate any cellular residents from arteries without altering extracellular support structures or overall vessel architecture^[69-72]^. This finding, though seemingly overlooked, could implicate the usage of PFA near arterial vessels, whose smooth muscle is critical to pressure regulation and subsequent flow.

Despite a decade of investigation, there is a lack of consensus regarding best practices to prevent inadvertent coronary injury. Many clinical practices now include the use of parenteral nitroglycerin during PFA in close proximity to coronary arteries, to reduce the risk of and sequelae from vasospasm^[73,74]^. This illustrates that short-and long-term reactive changes in cardiac vessels in response to PFA will continue to be a point of interest^[75]^. This portion of the review aims to succinctly summarize the preclinical and clinical ventricular PFA coronary data published to date.

### Preclinical coronary artery findings with ventricular PFA

The preclinical safety and efficacy studies evaluating ventricular PFA have been completed in *in vivo* swine and canine models of normal cardiovascular physiology or induced heart failure with reduced ejection fraction^[76-79,81-85]^ [Table 3]. The swine model remains the gold standard for evaluating clinical cardiac devices, given their similar cardiac dimensions, action potential physiology, and arrhythmogenic propensity compared to humans. Study durations ranged from 48 hours to 3 months. The most common method of coronary artery evaluation was direct visualization using contrast-enhanced fluoroscopy, while the most common method overall was histological analysis of coronary artery architecture. Of note, intimal hyperplasia secondary to coronary artery thermal injury is a slow, time-dependent process, and thus, these acute studies may underestimate the true incidence. Chronic studies to evaluate this phenomenon are needed.

Though the majority of studies were conducted with an epicardial approach by the same operators, the aggregate data^[76-79,81,83,85]^ support the conclusion of ventricular coronary arteries being relatively inert to PFA’s cytotoxicity in the immediate time course, as well as the short-term (days) and medium-term (months) time courses. It is necessary to acknowledge the limitation of these datasets with regard to study timeline duration: no chronic (years-long) studies exist, likely related to the prohibitive costs of housing, feeding, and instrumenting continually growing swine. Studies noted variable intimal hyperplasia in response to PFA without stenosis ^[76,78]^ and preserved nerve, artery, and vein architecture via histopathology^[81,85]^.

These findings are contrasted with more recent data from two distinct groups describing pathologic changes secondary to direct PFA^[82,84]^, though some involved methodologies that are not commonly used in clinical practice. The authors’ reports included acute coronary artery spasm secondary to both intra-coronary and epicardial PFA, as well as chronic stenosis via neointimal hyperplasia secondary to intra-coronary artery PFA [Table 4]. These findings are disconcerting in the context of the clinical use of nitroglycerin to prevent coronary artery vasospasm during PFA^[74,75]^, and duplication of these studies to corroborate the initial reports is paramount. A variable that may potentially account for these two divergent results is the location of PFA catheter placement. Though PFA is non-thermal in its cytotoxic mechanism of action, local heat generation is still theoretically possible, particularly with large voltages.

### Clinical coronary artery findings with ventricular PFA

Clinical data describing ventricular PFA are scarce, with only two case reports identified during an exhaustive search. The 2022 manuscript describes the use of PFA to treat myocardial infarction-related ventricular tachycardia in a patient who had undergone two prior attempts with radiofrequency ablation^[85]^. The authors report no acute complications from PFA, and the patient had no arrhythmia recurrence at 6 months post-procedure. The more recent case report^[86]^ documents the use of PFA for management of monomorphic VT and reports no evidence of electrocardiogram changes nor coronary artery spasm post-PFA (prophylactic 3 milligrams of intra-coronary nitrate was administered) as evidenced by angiography.

Though multiple clinical trials evaluating the safety and utility of PFA for atrial fibrillation substrate management have been completed, effort has yet to be undertaken to pursue a ventricular tachyarrhythmia equivalent. Additional challenges inherent to ventricular ablation include: ventricular wall thickness, motion from cardiac contraction and subsequent chamber diminution, as well as complex intracavitary structures.

### PFA coronary artery stenosis complication rate compared to thermal ablation

Given the limited datasets published on coronary artery injury secondary to PFA, its safety profile relative to thermal ablation remains unclear. Due to the non-thermal mechanism of action, it is reasonable to anticipate that the incidence of coronary artery stenosis will be lower for PFA compared to the thermal alternatives, but additional research is needed to support this hypothesis. Since PFA can damage vascular smooth muscle cells contained within muscular arteries, it must be presumed that both acute and chronic adverse structural changes may occur with this technology.

## CONCLUSIONS

Irreversible electroporation is a well-established molecular technique that has more recently been adapted to catheter-based technologies for use in invasive cardiac laboratories. The use of irreversible electroporation to modify cardiac arrhythmia substrates, termed pulsed field ablation (PFA), is increasingly represented in both preclinical and clinical studies, but is still in an early stage and has yet to be approved by the Food and Drug Administration.

Regarding atrial PFA applications, both preclinical and clinical data report no hemodynamically significant stenosis of pulmonary veins. The incidence of pulmonary vein stenosis may be lower for PFA compared to the thermal ablation modalities. This possibility is confounded by the lack of chronic studies available.

Regarding ventricular PFA applications, mixed preclinical data exist regarding whether PFA may be deleterious to coronary arteries. PFA, when applied with clinically appropriate pulse and field characteristics, is capable of ablating vascular cell populations, particularly smooth muscle, without disrupting non-cellular vessel architecture. Nonetheless, prophylactic intra-coronary nitro-based vasodilator administration likely mitigates the hemodynamic sequelae of this smooth muscle effect. Clinical data is purely observational at this present stage of ventricular PFA technology validation, with only one case study identified in the literature.

Future PFA preclinical studies should seek to corroborate prior *a priori* findings with appropriately powered cohort studies with chronic study durations, and future PFA clinical studies should incorporate high-resolution non-invasive cardiac imaging and functional testing to chronologically describe any coronary perfusion effects.

## Figures and Tables

**Table 1.** Preclinical Atrial pulsed Field Ablation Studies that Concluded No Pulmonary Vein Stenosis

Research Group *(Ref #)*	Year	Animal Model (Weight)	Sample Size	Study Design	Study Duration	Pulsed Field Ablation	Method of Stenosis Evaluation
Wittkampf (39)	2014	Swine (60-75 kg)	10	PFA *vs.* RFA	3 months	Endocardial; bipolar; 200J x 10	Angiograms; Histopathology
Reddy (40)	2019	Female Yorkshire Swine (60-70 kg)	17	Monophasic PFA *vs.* Biphasic PFA *vs.* RFA	10 weeks	Endocardial; Mono-800V x4 beats Bi-1800V x10 beats	Histopathology
Reddy (41)	2020	Female Yorkshire Swine (60-70 kg)	12	PFA: Low Dose **vs.** High Dose	4 weeks 2 weeks	Endocardial; 21-24A 24-28A	ICE
Su (42)	2021	Male Bama Miniswine (80±10 kg)	3	Bipolar PFA	3 days	Endocardial; 1600 V/cm @ 8A	Histopathology
Natale (43)	2022	Swine (no weights provided)	8	Safety: Supra-therapeutic PFA energy levels	1 month	Endocardial; 1800V	ICE; flow velocity
Zhang (44)	2022	Male & Female Swine (55 kg)	6	Biphasic PFA	1 month	Endocardial; 800-2000V	Histopathology
Reddy (45)	2023	Female Yorkshire Swine (no weights provided)	13	Single-shot PFA: Lose Dose *vs.* High Dose	1 week 5 weeks	Endocardial	Histopathology
Stewart (46)	2020	Male & Female Mongrel Hound Canine (28 kg)	8	PFA *vs.* RFA	12 weeks	Endocardial; 1500V	Cardiac CT
Reddy (47)	2023	Canine (29-36 kg)	29	PFA *vs.* RFA	0-30 days	Endocardial	Angiography

kg-kilograms. PFA-pulsed field ablation. RFA-radiofrequency ablation. J-joules. V-volts. A-amperes. CT-computed tomography. ICE-intracardiac echocardiography.

**Table 2.** Adult Clinical Atrial Pulsed Field Ablation Studies that Concluded No Pulmonary Vein Stenosis

Research Group *(Ref #)*	Year	Sample Size	Study Design	Follow Up Window	Pulsed Field Ablation	Method of Stenosis Evaluation
Reddy (48)	2018	22	1^st^ reported PFA use; catheter & surgical	1 month	Endocardial & epicardial; 900-2500V	ICE; voltage mapping
Reddy (49)	2019	81	Safety	120 days	Endocardial; monophasic (900-1000V) & biphasic (1800-2000V)	Electroanatomic mapping; Cardiac CT
Reddy (50)	2020	25	PVI + LAPW ablation for persistent AF	3 months	Endocardial; biphasic, bipolar; 1600-2000V	Cardiac CT
Reddy (51)	2020	80	PFA *vs.* RFA for PV stenosis	3 months	Endocardial; monophasic (900-1000V) & biphasic (1800-2000V)	Cardiac CT
Reddy (52)	2021	121	Durability of PVI	3 months	“Single shot” endocardial; 1600-1800V	Cardiac CT
Schmidt (53)	2022	20	PFA with CIEDs	None	“Single shot” endocardial; biphasic; 1900-2000V	Angiography
Willems (54)	2022	15	Repeat AT ablation after prior AF/AT ablation	None	Endocardial; bipolar; 2000V	Angiography
Reddy (55)	2023	21	Safety and Durability of “Single Shot”; single *vs.* triple dose	3 months	Endocardial; 1700V	Electroanatomic mapping
Schmidt (56)	2023	360	Repeat PVI with 31*mm vs.* 35*mm* catheter	6±4 months	Endocardial; 1800-1900V	Angiography
Reddy (57)	2023	226	Safety and Durability of PVI	3 months	Endocardial; bipolar, biphasic; 1800V	Cardiac CT

PFA-pulsed field ablation. ICE-intracardiac echocardiography. CT-computed tomography. V-volts. PVI-pulmonary vein isolation. LAPW-left atrial posterior wall. AF-atrial fibrillation. PV-stenosis. CIED-cardiac implantable electronic device. AT-atrial tachycardia.

**Table 3.** Preclinical Ventricular Pulsed Field Ablation Studies that Concluded No Coronary Artery Injury

Research Group *(Ref #)*	Year	Animal Model (Weight)	Sample Size	Study Design	Study Duration	Pulsed Field Ablation	Method of Stenosis Evaluation
Wittkampf (71)	2013	Swine (60-75 kg)	9	Lesion *vs.* non-lesion coronary dimensions	3 weeks	Epicardial; monophasic; 30-360J	Histopathology
Wittkampf (72)	2014	Swine (60-75 kg)	5	Lesion *vs.* non-lesion coronary dimensions	3 months	Epicardial; monophasic; 200J	Angiography; Histopathology
Wittkampf (73)	2014	Swine (60-75 kg)	5	PFA Dose-Response	3 months	Epicardial; monophasic; 50-200J	Histopathology
Wittkampf (74)	2014	Swine (60-75 kg)	5	PFA Dose-Response	3 months	Epicardial; monophasic; 30-300J	Angiography; Histopathology
Gerstenfeld (75)	2022	Female Swine (65.5 kg)	10	Lesion Qualities: PFA *vs.* RFA	2 hours	Endocardial; biphasic, bipolar; 2000V	Histopathology
Wittkampf (77)	2023	Female Swine (60-75 kg)	6	PFA in Coronary Sinus	3 weeks	Endocardial; monophasic; 100J	Angiography; Histopathology
Asirvatham (79)	2022	Mongrel Canine (30-45 kg)	8	PFA across IVS	30 days	Endocardial; bipolar; 1000-1500V	Angiography; Histopathology

kg-kilograms. PFA-pulsed field ablation. J-joules. PFA-pulsed field ablation. RFA- radiofrequency ablation. V-volts. IVS-interventricular septum.

**Table 4.** Preclinical Ventricular Pulsed Field Ablation Studies that Concluded Yes Coronary Artery Injury

Research Group *(Ref #)*	Year	Animal Model (Weight)	Sample Size	Study Design	Study Duration	Pulsed Field Ablation	Method of Stenosis Evaluation
Asirvatham (76)	2022	Swine (no weights provided)	14	IC and Epicardial PFA	1 month	Bipolar or Unipolar; 10-143J	Angiography; Histopathology
Gerstenfeld (78)	2022	Female Yorkshire Swine (55-65 kg)	4	Direct Coronary PFA	8 weeks	Epicardial; 2000V	Angiography; Histopathology

kg-kilograms. IC- intra-coronary. PFA-pulsed field ablation. J-joules. V-volts.

## Data Availability

Not applicable.
